# Image: Colonic Hematoma Complicating Pediatric Colonoscopy

**DOI:** 10.1097/PG9.0000000000000128

**Published:** 2021-10-25

**Authors:** Chetan Mandelia, Kaliyann Te, Mary Lenfestey

**Affiliations:** Division of Pediatric Gastroenterology East Carolina University Brody School of Medicine Greenville, North Carolina

**FIGURE 1.**
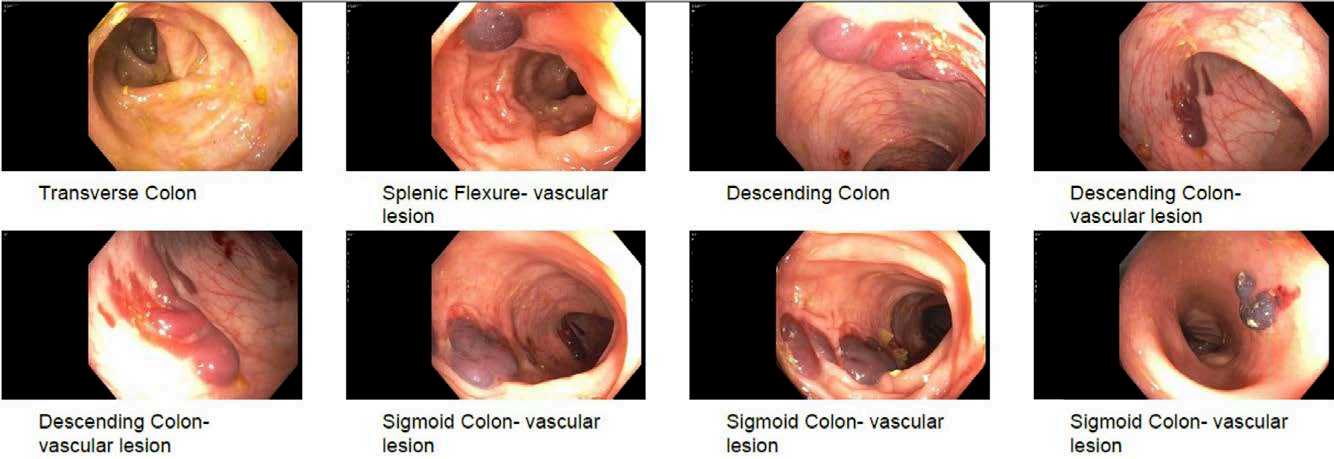
Colonic vascular lesions. Four discreet lesions seen in splenic flexure, descending colon and sigmoid colon (×2).

A 6-year-old girl with chronic constipation was referred for intermittent mass per rectum. Colonoscopy was performed to exclude rectal polyp. Due to poor bowel preparation and looping of colonoscope in the redundant sigmoid colon, the visualization was poor as the scope was advanced through the colon and procedure was prolonged but successfully completed. On withdrawal of the colonoscope, 4 discreet vascular lesions were seen between splenic flexure and sigmoid colon (Fig. 1). The lesions were raised, purple in color, interspersed with normal colonic mucosa, and not actively bleeding. The findings were thought to represent angiodysplasia with concern for blue rubber bleb nevus syndrome (1). Coagulation studies, computerized tomography angiogram chest/abdomen, esophagogastroduodenoscopy, and capsule endoscopy were subsequently normal. Repeat flexible sigmoidoscopy 3 months later was normal; the previously visualized lesions were not seen. The vascular lesions were most likely intramural hematomas resulting from trauma secondary to looping of colonoscope and loop reduction maneuvers.

Colonic intramural hematoma is an uncommon complication of diagnostic colonoscopy, with only a handful of reported cases (2–4). Colonic hematomas have been reported in children secondary to accidental and nonaccidental trauma, bleeding disorders, and anticoagulant therapy (5–8). This is one of the first reported case of colonic hematoma complicating diagnostic colonoscopy in a pediatric patient. No cases of colonic hematoma have been reported in the Pediatric Endoscopy Database System-Clinical Outcomes Research Initiative database (personal communication from Dr Douglas Fishman, June 15, 2021). In our case, the knowledge gap due to lack of reported cases and images of this complication led to an extensive evaluation for angiodysplasia, which could have been avoided if the hematoma had been correctly identified. The parents consented to publication of the details of this case.
